# Efficacy and Animal Welfare Impacts of Novel Capture Methods for Two Species of Invasive Wild Mammals in New Zealand

**DOI:** 10.3390/ani10010044

**Published:** 2019-12-24

**Authors:** A. David M. Latham, Ben Davidson, Bruce Warburton, Ivor Yockney, Jordan O. Hampton

**Affiliations:** 1 Manaaki Whenua–Landcare Research, P.O. Box 69040, Lincoln 7640, New Zealand; warburtonb@landcareresearch.co.nz (B.W.); yockneyi@landcareresearch.co.nz (I.Y.); 2 Rangiora Vet Centre, Rangiora 7471, New Zealand; ben@rangvet.co.nz; 3 School of Veterinary and Life Sciences, Murdoch University, 90 South Street, Murdoch, Perth 6150, Australia; jordan.hampton@murdoch.edu.au

**Keywords:** Bennett’s wallaby, *Cervus elaphus*, darting, ground-based netting, net-gunning, *Notamacropus rufogriseus*, red deer, thiafentanil, tiletamine–zolazepam

## Abstract

**Simple Summary:**

The animal welfare impacts of wildlife capture methods are an important contemporary issue in conservation. We assessed novel methods for capturing Bennett’s wallabies (*Notamacropus rufogriseus*) and red deer (*Cervus elaphus*), both invasive mammals in New Zealand. The methods assessed were helicopter net-gunning for both species, versus ground-based netting for wallabies and helicopter darting for deer. We quantified efficacy, duration of procedures, frequency of adverse events, and cost-effectiveness for each method. We discuss the benefits of our approach for assessing new wildlife capture methods.

**Abstract:**

All capture methods impose animal welfare impacts, but these impacts are rarely quantified or reported. We present data from two wildlife capture studies that trialled new methods for capturing Bennett’s wallabies (*Notamacropus rufogriseus*) and red deer (*Cervus elaphus*) in New Zealand. We used helicopter net-gunning for both species, and compared this method with ground-based netting for wallabies and helicopter darting for red deer, using, for the first time in New Zealand, the fast-acting opioid thiafentanil. Efficacy and animal welfare parameters quantified were duration of handling and recovery, and frequency of adverse events, including escape, injury, and mortality. Cost-effectiveness was quantified for each method. Capture mortalities occurred for all methods for both species. For red deer, chemical immobilisation led to fewer traumatic injuries and fewer mortalities, while for wallabies, net-gunning led to fewer mortalities. Net-gunning was an efficient capture method for deer in open habitat, but led to the escape of 54% of wallabies and one wallaby mortality (4%). Ground-based netting resulted in the mortality of 17% of wallabies at the time of capture, and the capture of non-target species. The cost per captured wallaby was 40% more expensive for net-gunning (NZ$1045) than for ground-based netting (NZ$745), but, once corrected for mortalities at the time of capture and suitability of individuals for GPS-collar deployment, this was reduced to 29% and 12% more expensive, respectively. Net-gunning for red deer resulted in the escape of 13% of animals and mortality of 10% of animals at the time of capture. Helicopter-based darting for red deer using thiafentanil (c. 0.03–0.06 mg/kg) had high capture efficacy (zero escapes), rapid induction times (mean of 3 min), and a low mortality rate at 14 days post-capture (3%), but it was more expensive per deer captured and collared than aerial netting (NZ$2677 and NZ$2234, respectively). We recommend reporting of adverse event data for all wildlife capture techniques to permit continual refinement of field methods.

## 1. Introduction

Introduced terrestrial vertebrates can have significant unwanted impacts in native forests and production landscapes [1,2]. Bennett’s wallabies (also known as red-necked wallabies, *Notamacropus rufogriseus*) and red deer (*Cervus elaphus*) were introduced into New Zealand in the late 1800s [3,4]. The distribution of Bennett’s wallabies has continued to expand from their point of release in the Hunters Hills in South Canterbury, South Island. They are common within a continuous distribution of c. 5000 km^2^, but are believed to occur over a much larger, often discontinuous, range of c. 15,000 km^2^ [5]. Red deer are widespread and common on both main islands [4]. Both species can have significant unwanted impacts on forests and pasture [6,7,8], especially at high densities (c. 300 or more wallabies/km^2^ [3] and c. >15 deer/km^2^) [9], but also at lower densities for plant species that are highly vulnerable to their browsing [9].

To mitigate the unwanted impacts of Bennett’s wallabies, Environment Canterbury (the management agency responsible for biosecurity in this region) has delineated a ‘containment area’ with the aim of preventing their spread from this area and reducing their densities within it using various methods of lethal control [3]. Recently, however, wallabies have ‘escaped’ from the containment area via natural spread and illegal liberations, necessitating the involvement of neighbouring biosecurity agencies whose regions have been invaded [5].

Red deer have been managed as pests in New Zealand since c. 1930 [4]. Initially deer control was conducted by government-employed ground hunters, but this form of culling was only effective at reducing deer populations in the most accessible areas [10]. The management of wild deer (and other wild ungulates) in New Zealand is currently the responsibility of the New Zealand Department of Conservation (DOC). DOC uses a site-based prioritisation process to identify key conservation sites, and includes deer control at a relatively small number of these sites where deer are a key threat [4]. Outside of these areas, DOC relies on commercial and recreational hunters to reduce wild deer populations and their unwanted impacts [4].

In concert with lethal control or harvesting of Bennett’s wallabies and red deer, there is a need for improved science-based approaches for managing these species, especially with new technologies such as thermal-imaging cameras and drones becoming more available and affordable. These types of studies often require wildlife capture and marking using various types of animal-borne electronic sensors, such as GPS-collars [11]. However, all capture methods impose animal welfare impacts. Macropods (which includes wallabies) are known to be highly susceptible to stress-related diseases such as capture myopathy when restrained for attaching sensors [12,13,14]. Capture-related stress has also been widely documented for red deer and is affected by capture method, with physical restraint usually shown to be more stressful than chemical immobilisation [15,16]. Therefore, any research requiring the capture and deployment of animal sensors needs to consider the efficacy and animal welfare impacts of the methods used.

The importance of animal welfare in wildlife management is receiving ever-increasing recognition. While most animal welfare research has focused on lethal techniques used to harvest and cull animals, there is growing interest in improving our understanding of the effects of non-lethal methods, such as capture methods used to study, rather than manage, wildlife populations [17,18]. There is a growing requirement for wildlife researchers to report on the animal welfare outcomes of the capture methods they use, whether they demonstrate negligible or substantial animal welfare impacts [19]. A variety of capture methods have been used to capture wild macropods, including darting [20], pole syringes [21], draw-string traps [22], stunning using a closely placed shot from a high-velocity centrefire rifle [23], and medicated bait [24].

There is a long history of live-capturing wild red deer, especially for obtaining stock to meet the demand from the burgeoning deer farming industry in New Zealand in the late-1970s to mid-1980s [25], using helicopter-based darting, net-gunning and bulldogging (jumping out of a helicopter onto the deer’s back and wrestling it to the ground) [25]. However, many wildlife capture techniques previously used in New Zealand are now considered antiquated (e.g., stunning and medicated bait for wallabies, and bulldogging for red deer), and authors have often failed to adequately describe the frequency of adverse events (especially mortalities and injuries) associated with the method used. Poorly understood aspects of capture techniques include efficacy, cost-effectiveness, and animal welfare impacts.

Helicopter net-gunning is an important capture technique for many species of medium-sized mammals, such as bighorn sheep (*Ovis canadensis*, [26,27]) and canids (wolves, *Canis lupus*, and coyotes *C. latrans*, [28,29]). Net-gunning has also been widely used for capturing red deer in New Zealand since about the late 1970s [25,30]. However, we are unaware of any studies attempting to capture any species of marsupial using helicopter net-gunning. Similarly, we are unaware of any published studies from New Zealand, or elsewhere, that have used the fast-acting opioid thiafentanil for darting red deer from a helicopter. However, the fast onset of effects associated with this opioid, comparatively short time to recumbency, and minimal effects on heart and respiratory rates suggest it may have high efficacy and low risks for the welfare of darted animals relative to many other methods of capture [31].

For this reason, thiafentanil combinations have been used in several recent helicopter darting studies (e.g., for water buffalo, *Bubalus bubalis*, in northern Australia) [32]. Like all capture methods, net-gunning for macropods and darting using thiafentanil for deer will have logistical constraints, risk of injury or death to operators, and risk and unwanted side-effects for the immobilised animal. However, the unwanted effects associated with new capture methods must be compared with those of existing capture methods, as they could have improved efficacy and animal welfare outcomes.

Here we document the first attempt to capture macropods using net-gunning (a net fired from a gun) from a helicopter and compare the efficacy and animal welfare impacts of this method with physically restrained and chemically immobilised Bennett’s wallabies captured using ground-netting (i.e., a more traditional capture method) [33]. We also document helicopter-based darting for red deer using thiafentanil for the first time in New Zealand. We did not trial helicopter-based darting for wallabies due to the difficulties associated with accurately placing a dart in a relatively small animal from a moving platform. We compare the efficacy and animal welfare impacts of darting deer with those of net-gunning deer, both from a helicopter. These capture techniques were chosen as being appropriate to the rugged terrain of the field sites. Trapping has been used for capturing some species of deer but would be impractical in alpine areas.

The efficacy and animal welfare parameters that we quantified were duration of handling and recovery, and frequency of adverse events, including escape, injury and mortality. The animal welfare impact data collected were peripheral to the main objectives of the studies, but both studies provided sufficient data to allow an initial examination of the animal welfare impacts of the live-capture methods used. We also assessed cost-effectiveness for each capture method.

## 2. Methods

### 2.1. Wallaby Study Sites

We captured and GPS-collared adult wallabies with the aim of estimating detection probabilities for a range of ground-based and aerial survey methods to improve the management of wallabies in New Zealand (Latham and Warburton, unpubl. data). Adult Bennett’s wallabies weigh about 9–18 kg, but occasionally up to 25 kg [3]. The GPS-collars we used were customised to enable fast fix rates (i.e., to record a location every 5 s from 6:30 h to 12:00 h when surveys for wallabies were conducted) and weighed c. 200 g. We only collared adult wallabies (≥9 kg), and so the weight of the collars did not exceed about 2% of wallaby body weight, within the accepted international standard [11,34]. The capture and collaring protocols for wallabies were approved by the Manaaki Whenua–Landcare Research (MWLR) Animal Ethics Committee (AEC) (approval No. 17/11/03).

**Figure 1:**
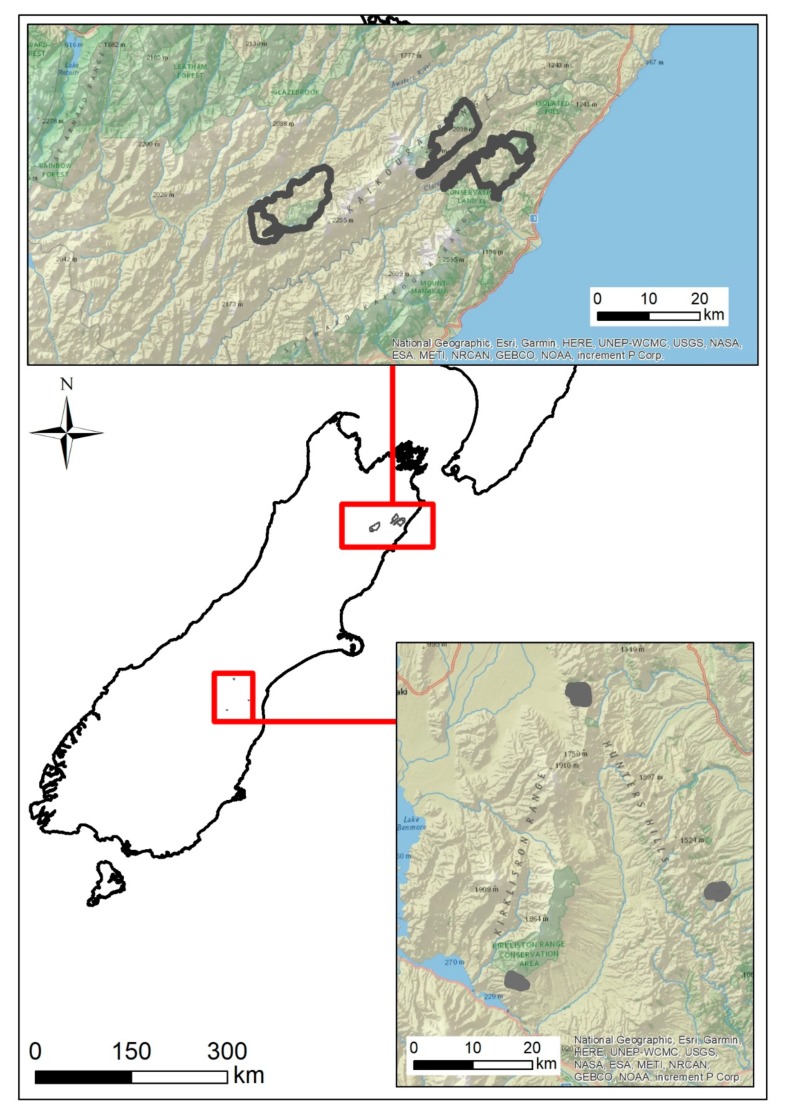
Map of study sites for two wildlife management studies performed on the South Island, New Zealand. Bennett’s wallabies (*Notamacropus rufogriseus*) were captured in the southern site, South Canterbury at three different high-country stations (farms) during 2018–2019. Red deer (*Cervus elaphus*) were captured at the northern site, near the Inland Kaikōura Range, south Marlborough in 2019.

We captured wallabies at three different high-country stations (farms) farming sheep and beef cattle in South Canterbury (Figure 1). All three stations were located within the wallaby containment area delineated for Bennett’s wallaby by Environment Canterbury [5].

Our first trial took place at Glen Cary Station, Waimate District, in the southern part of the containment area, in May 2018. Glen Cary is characterised by pasture at lower elevations, and tall snow tussocks (*Chionochloa* spp.) and matagouri (*Discaria toumatou*) scrub at higher elevations. Our second trial took place at Blue Cliffs Station, Waimate District, in the eastern central part of the containment area, in August 2018. Blue Cliffs is a mosaic of pasture, matagouri scrub, snow tussocks, and patches of remnant native forest and scrub. Our third trial took place at the Grampians Station, Mackenzie District, in the western central part of the containment area, in February 2019. The Grampians was the most open study site, comprising mostly short and tall tussocks, with some matagouri scrub.

Study sites ranged from c. 500 to 900 ha in size. The climate at all three sites was dominated by dry (annual rainfall = c. 300–600 mm), temperate, continental conditions.

### 2.2. Helicopter Net-Gunning Procedures for Wallabies

**Figure 2:**
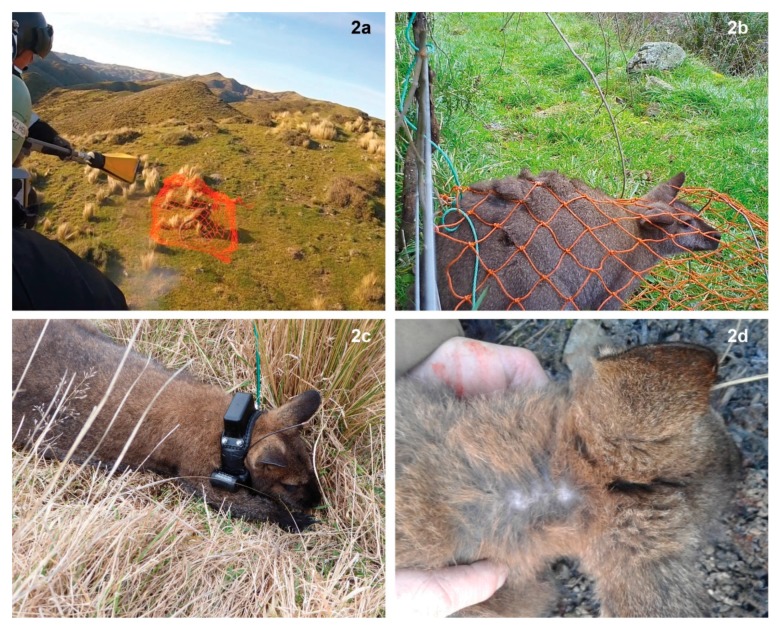
Methods used for capturing Bennett’s wallabies (*Notamacropus rufogriseus*) in South Canterbury, New Zealand, 2018–2019, included (**a**) helicopter-based net-gunning and (**b**) ground-based netting. The GPS-collars we used (**c**) were customised and weighed c. 200 g: we only collared adult wallabies (≥9 kg), and therefore the weight of the collars did not exceed about 2% of wallaby body weight, within the accepted international standard. The GPS-collars caused only minor rubbing (**d**) of the neck fur on collared individuals, even after they were worn for 1 to 2 months.

We used net-guns fired from the front right seat of an MD520N helicopter (MD Helicopters, Mesa, AZ, USA) to capture adult wallabies in open or semi-open habitats, including pasture, tussock and scrub interspersed with clearings (Figure 2a). The net-gun used was a Wildlife Capture Equipment^®^ WCE net launcher (Wildlife Capture Equipment, Flagstaff, AZ USA; www.wildlifecaptureequipment.com/) firing 10′ × 10′ knotted flat braid nets of 4″ mesh size. The net-gun was charged with a 0.308 Winchester blank cartridge and we carried four spare pre-packed nets while undertaking capture. The effective capture range of net-guns for wallabies was 5–15 m, but most wallabies were captured in a line below horizontal from the shooter’s position at 5–10 m. A tail boom-mounted digital video camera (GoPro^®^, San Mateo, CA, USA) enabled us to quantify chase time (CT) when video footage was reviewed after field operations. We defined CT as the interval between a wallaby first being pursued with the helicopter and the net-gun being fired [35].

### 2.3. Ground-Based Netting for Wallabies

We set ground-based nets at wallaby runs (i.e., well-used trails) under farm fences (Figure 2b). Ground-based “tunnel” nets were made of nylon string woven into a diamond-mesh of c. 4 cm × 6 cm. The nets were c. 1–1.2 m in length, conical in shape, and the net entrance was slightly oval (c. 40–50 cm high × 50 cm wide). The mouth of the net was fixed at four to five places on the lower two strands of wire on fences using a wire clip that was designed to pop off the fence if pressure was applied in the net. A semi-circular hoop made of no. 8 gauge wire and pegs at the base of the net were used to keep the set net taut. Once a wallaby entered the net and applied pressure to it, the net popped off the fence and the entrance of the net was drawn tight by a drawstring, leaving the wallaby trapped inside. An anchor line attached to the fence prevented the entangled wallaby from moving away from the point of capture.

We attempted to check all nets between 7:00 h–12:00 h, but occasionally a small number of nets were checked in the early afternoon. Any nets that had collapsed or been flattened were reset by a field technician after they had been checked for a captured wallaby, while one or two field staff moved to the next set net to check it.

To monitor the behaviour of wallabies captured in ground-based nets, we erected Reconyx^®^ Hyperfire^TM^ PC900 camera traps (Reconyx, Holmen, WI, USA) at 12 set nets. We programmed cameras to take five photos in rapid succession (1 s delay between photos) during the day and the night when activated by a moving animal.

### 2.4. Wallaby Handling

We blindfolded captured wallabies and measured body temperature using a rectal thermometer. Two methods were used for processing captured animals: chemical immobilisation and physical restraint. We used tiletamine–zolazepam (500 mg powder; Zoletil^®^; Virbac, NSW, Australia), applied as an intramuscular injection at a dose not exceeding 4 mg/kg to sedate captured wallabies [36]. We sedated every second adult wallaby captured on Blue Cliffs to compare animal welfare outcomes for wallabies that received chemical restraint vs. physical restraint. We monitored sedated wallabies until they had fully recovered and were standing, as per standard immobilisation practice. This permitted the recording of recovery time for sedated animals.

Because there is no commercially available specific antagonist (reversal agent) for the tiletamine component of tiletamine–zolazepam, the processing and recovery time took too long (>30 min) to be compatible with helicopter use, so we only used physical restraint for all net-gunned wallabies. We restrained wallabies by having one assistant firmly hold their strong (and potentially dangerous) back legs and one assistant apply lighter pressure at the shoulders, while a field technician attached a bespoke GPS-collar (Figure 2c).

### 2.5. Red Deer Study Site

We captured red deer and fitted them with VHF mortality-sensing collars to monitor incidental deer by-kill during an aerial poison operation delivered for the lethal control of brushtail possums (*Trichosurus vulpecula*) (see [37]). The research was conducted for OPSRI NZ to test a new deer-repellent bait. The efficacy of the repellent was assessed by comparing the frequency of mortality of the collared red deer in an area treated with repellent bait (13,280 ha), with the frequency of mortality in an area treated with standard non-deer-repellent bait (9447 ha). The sites chosen were in south Marlborough in two large adjacent valleys, with the Inland Kaikōura Range dividing the two valleys (Figure 1). The Clarence Valley (Clarence West/Waiautoa) had the deer-repellent treatment applied, while the Awatere Valley (Upper Awatere South) site was the non-treatment area.

Land tenure was a mix of private and public conservation land, with the altitude of deer capture varying between 200 and 2000 m above mean sea level (AMSL) in Clarence West/Waiautoa and 800–1600 m AMSL in Upper Awatere South. Upper Awatere South site is an un-forested high-country merino sheep-grazing station of semi-arid habitat (<700 mm rainfall per annum), with vegetation consisting of improved grasses, short and tall tussocks, sweet briar (*Rosa rubiginosa*), matagouri, and subalpine species [38]. Clarence West/Waiautoa is a mix of public conservation estate at higher altitudes, with beech forest and alpine vegetation and private land of regenerating scrubland on lower elevations. Such areas, particularly on the Waiautoa block were heavily dominated by mānuka (*Leptospermum scoparium*) and kānuka (*Kunzea ericoides*), matagouri, sweet briar, and mixed native forest, interspersed with grass clearings of pasture. Mean annual rainfall for this area is around 800 mm per annum (source: NIWA Climate Summaries; https://niwa.co.nz/climate/summaries). Both sites were considered steep and challenging for helicopter operations and safe live capture of deer.

### 2.6. Red Deer Capture

We used both physical restraint (net-gun) and chemical immobilisation (dart gun) to capture deer and deploy VHF radio-collars on them. We endeavoured to catch an even ratio of adult males and females and sub-adult/juveniles as part of the research. Capture took place in late autumn between 10 May 2019 and 19 June 2019 and was conducted under optimal weather conditions, which were largely determined by calm wind conditions in order to safely manoeuvre the helicopter for this type of work. We used an MD520N due to its manoeuvrability and power, which are key requirements for low-level flying.

The capture team consisted of the pilot, a wildlife researcher (shooting dart and net gun), and a veterinarian. For this live-capture work we operated the helicopter with all doors off for better visibility and to allow ease of egress for shooter and veterinarian on steep terrain. We spotted deer visually, and the pilot, shooter (net-gun and dart gun), and veterinarian selected deer suitable for capture and determined the most appropriate capture method. Capture method was largely determined by the sex and age of the targeted individual animal and the surrounding topography and vegetation. Because stags were still in hard antler, we had previously decided not to net any mature stags due to capture and handling difficulty. The second key decision we made was not to dart deer near mature forest or thick vegetation that would impinge on our ability to watch the darted animal through all stages of post-dart chemical immobilisation. While we did not predetermine a CT limit (as per [35]), chases were called off (terminated) if animals approached the block boundaries, entered into difficult terrain that would make capture difficult for targeted individuals or capture crew, or if deer appeared severely distressed (open-mouth breathing, losing their footing, colliding with obstacles) and we believed the capture process would negatively affect the animal’s welfare. Deer were monitored by aerial radio tracking in fixed-wing aircraft at regular intervals on seven occasions between their initial capture date and project completion, which ranged from 1–3 months post-capture.

### 2.7. Helicopter Net-Gunning Procedures for Red Deer

**Figure 3:**
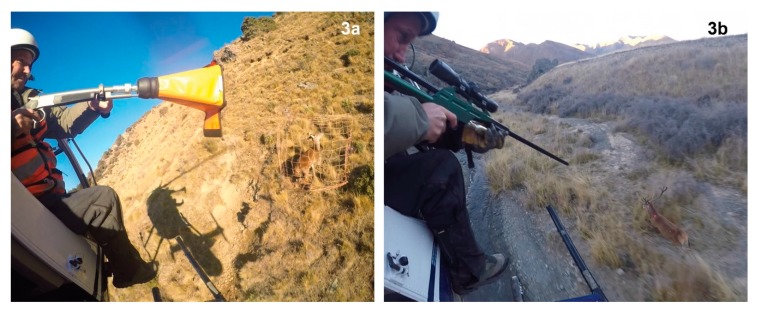
Methods used for capturing red deer (*Cervus elaphus*) in the Inland Kaikōura Range, south Marlborough, New Zealand, 2019, included (**a**) helicopter-based net-gunning, and (**b**) helicopter-based darting using the pharmacological agent thiafentanil.

We used net-guns fired from the front right seat of an MD520N helicopter to capture red deer once an individual animal was targeted (Figure 3a). The net-gun used was a Wildlife Capture Equipment^®^ WCE net launcher firing 12′ × 12′ knotless Dyneema^®^ nets of 7″ mesh size. The net-gun was charged with a medium load 0.308 Winchester blank cartridge, and we carried four spare pre-packed nets while undertaking capture. The effective capture range of net-guns for deer was the same as for wallabies (5–15 m), with most deer being captured in a line below horizontal from the shooter’s position at 5–10 m. A capture zone was considered appropriate for net capture when there was little chance of the animal falling uncontrollably, and the immediate area was clear of vegetation that could hold a net up but where the animal would tangle well in nearby vegetation. Duration parameters were quantified for capture events by reviewing video footage from a wearable digital video camera, as for wallaby net-gunning (with the difference being that the video camera was worn inside the helicopter for red deer capture and was attached to the exterior of the helicopter for wallabies).

### 2.8. Helicopter Darting Procedures for Red Deer

For chemical immobilisation (Figure 3b), we used a Dan-Inject^®^ JM SP25 dart projector with an 11 mm diameter barrel firing 3 mL pressurised darts with 2 mm × 30 mm (diameter × length) barbed needles (Dan-Inject Australasia, Harcourt, Australia; https://daninject.com.au/). We preloaded darts with 4.0 mg thiafentanil for female deer and 5.0 mg thiafentanil for male deer in Upper Awatere South, whereas we used a standard dose of 4.5 mg for all deer in Clarence West/Waiautoa. Our rationale for using a standard dose of 4.5 mg in Clarence West/Waiautoa was that there was substantial overlap in the weight of males and females, especially between study sites, and as both doses had already proven effective in Upper Awatere South, it was simplest to use an intermediate standard dose. This equated to a mean dose of 0.038 mg/kg and 0.034 mg/kg for female and male deer (respectively) in Upper Awatere South and a mean dose of 0.064 mg/kg and 0.047 mg/kg for female and male deer, respectively, for the Clarence West/Waiautoa deer. Each dart also contained the enzyme hyaluronidase [36] with a standard 1500 IU dose (c. 19 IU/kg). Darts were pre-loaded, pressurised, and stored in a secure box in the front of the helicopter accessible by pilot and shooter, along with two syringes of the human reversal agent naltrexone, as per [39].

Pursuit and capture were performed using an MD520N helicopter, with darting distances estimated at 5–20 m. Capture zones were not considered as for net capture, due to the distance deer run during induction with darting. We attempted to dart intramuscularly in the neck, shoulder, back or rump; however, some deer were darted subcutaneously in other parts of the body due to the difficulty associated with the effects of rotor wash on the trajectory of the darts. If a deer was subcutaneously darted, we fired a second dart or a net-gun to complete the capture. Once a dart had been fired and lodged in the animal, the veterinarian seated in the back of the helicopter started a stopwatch to record all phases of anaesthesia and recovery. We recorded induction time (IT) and recumbent time (RT) [40], and for a smaller subset of deer, CT (i.e., where the full chase was recorded on video). Reversal was given in the form of naltrexone at 50–75 mg depending on whether the animal received one dart or more (c. 0.6 mg/kg).

### 2.9. Red Deer Handling

Once captured, deer were quietly approached by the veterinarian and wildlife researcher and immediately blindfolded to quieten the animal. Deer were adequately restrained (if in a net) and positioned appropriately so as not to encumber breathing (if sedated). Manual restraint was not required for darted deer. We fitted all deer with a mortality-sensing remote drop-off VHF collar weighing 550 g (Lotek Wireless Inc., Havelock North, New Zealand). Captured deer were assessed by the veterinarian before being untangled and released from the net or given a reversal dose of naltrexone. We captured 13 deer by net-gunning without a veterinarian present, utilising two wildlife research staff instead. 

## 3. Results

### 3.1. Wallaby Demography

All 11 wallabies captured using a net-gun fired from a helicopter were adult males. Selection for males was unintentional. The weights of these individuals were not recorded. Fourteen of 36 wallabies captured using ground-based trapping were male, 21 were female and one was unknown. The mean estimated or measured weight of 34 ground-trapped wallabies was 11 kg (4–15 kg), marginally lower than the mean for the 27 wallabies on which we deployed GPS-collars (12 kg; range = 9–15 kg).

### 3.2. Helicopter Net-Gunning for Wallabies

We used net-gunning at the two study sites (Glen Cary and the Grampians) with the most open habitat. We caught and GPS-collared 11 of 24 wallabies that were netted (54% escape rate; Table 1). The 13 wallabies that were netted but not collared either managed to slip out of the net before they became tangled in it or before capture personnel could get to them and restrain them. The mean helicopter CT for 11 wallabies (six at Glen Cary and five at the Grampians) at which a net was fired was 34 s (range = 5–89 s). All helicopter-captured wallabies were in open habitat (tall tussock, short tussock, bare ground, and open matagouri scrub). All wallabies caught with net-guns were physically restrained and handling times were estimated to be between 3 and 5 min. The primary reasons for wallabies that were seen from the helicopter evading capture were targeted individuals that reached cover (scrub or remnant forest) before a net could be fired at them, or the net missed them, usually because a poor shot was offered, or the wallaby changed direction suddenly.

No wallabies captured using a net-gun suffered any form of obvious external injury at the time of capture. Ten collared wallabies captured by net-gunning remained alive at the completion of our study; apart from minor rubbing of the neck fur by the collar (Figure 2d), none of these animals had noticeable injuries. One wallaby died a short-time (<5 min) post-capture from net-gunning (i.e., it was found lying in an open area and on checking was found to be dead). The cause of death was inferred to be capture myopathy [41], possibly due to hyperthermia, as capture staff believed the CT for this animal was longer than for other netted individuals (the CT for this individual was not recorded on video camera, but was probably greater than 2 min). However, the body temperature of this individual (35.3 °C) was intermediate within the observed range of temperatures (34.4–37.0 °C) for other helicopter-chased wallabies, albeit based on a sample of only four individuals. In consultation with our Animal Ethics Committee, we attempted to mitigate this problem by reducing CT to a maximum of 2 min; this time includes the helicopter trying to haze the targeted animal out from underneath scrub (i.e., hovering at close proximity to elicit a flight response) so a net can be fired at it.

### 3.3. Ground-Based Netting for Wallabies

We used ground-based netting at all three study sites, and it was most effective when used along fence lines in pasture, tussock, scrub, and forest. We captured 36 wallabies using this method, 26 of which were fitted with a GPS collar. Total mean trapping success per net per day was 0.07 wallabies (range = 0.05–0.12); the maximum number of nets set at any one time was 83 (mean = 56). It took approximately 5 h (c. 7:00 h–12:00 h) and 8 h (c. 7:00 h–15:00 h) to check 56 and 83 nets, respectively; checking times were dependent on how many wallabies were captured each day.

Based on camera trap footage, one wallaby that entered a ground-based net managed to turn around in it and escape. To our knowledge no other wallabies managed to escape once they had entered a net; however, set ground-based nets were often found trampled flat by wallabies, especially if they were set near a wallaby trail running parallel to the fence.

Ground-based netting had a mortality rate of c. 17% (*n* = 6) at the time of capture (Table 1). Causes of these deaths were: probable strangulation in the net (i.e., the head was through the net and it was wrapped tightly around the throat; *n* = 3); probable sciatic nerve damage (*n* = 1; subsequently euthanased); probable exhaustion (*n* = 1); and unknown cause (*n* = 1). Also, three wallabies had minor abrasions to the tail or hind leg. At the end of the study (c. 2–3 months later), a total of 11 wallabies (31%) captured in ground-based nets were dead. The causes of post-capture deaths are unknown; however, GPS data showed that one wallaby died c. 1 week post-capture and therefore this death may be attributable to capture myopathy (i.e., the individual appeared exhausted at the time of capture). All other deaths occurred >2 weeks after capture and were not attributed to capture. Based on an assessment at the time collars were retrieved, wallabies suffered only minor rubbing of the neck fur by the GPS collar (Figure 2d).

Ten captured females had dependent pouch-young. One captured adult female that was processed using physical restraint had her pouch young (estimated to be c. 5–6 months of age) ejected from the pouch during handling and this juvenile was subsequently euthanased. All other young (*n* = 9) remained in the pouch during the handling process.

Three of 12 camera traps set up to view captures of wallabies at ground-based nets collected usable data on confinement time (total time the wallaby was confined in the net) and struggle time (the amount of time the wallaby spent struggling while it was confined). All three wallabies entered nets in the evening and were in them until the nets were checked the following day. The total confinement times for these wallabies were 12 h 55 min, 14 h 39 min, and 19 h 01 min. The respective total cumulative struggle times for these three individuals were 5 min, 21 min and 41 min.

Two of these individuals died. One individual (adult female) was confined for almost 13 h and suffered probable permanent nerve damage and was euthanased (see above). The individual (adult male) confined for the greatest period and that struggled most while in the net was unresponsive when capture staff arrived; this animal had the lowest recorded body temperature (33.6 °C) of any measured. It was found dead approximately 2 weeks post-capture. GPS location data showed it had not moved far from its post-capture release site, suggesting it never fully recovered from capture. Struggle time was not related to how long an animal was confined (i.e., our small sample showed that all three captured animals struggled within the first hour after being trapped in the ground-based net, but not thereafter).

We used tiletamine-zolazepam to chemically immobilise 10 ground-netted wallabies [35] and we physically restrained 22 captured wallabies (the remaining four ground-captured wallabies were found dead in nets). There were no cases of capture myopathy for chemically immobilised animals; two physically restrained animals subsequently died, but these deaths were probably not related to physical restraint by capture staff (i.e., one suffered sciatic nerve damage from the net and one had been confined in the net for 19 h; both described above).

**Figure 4:**
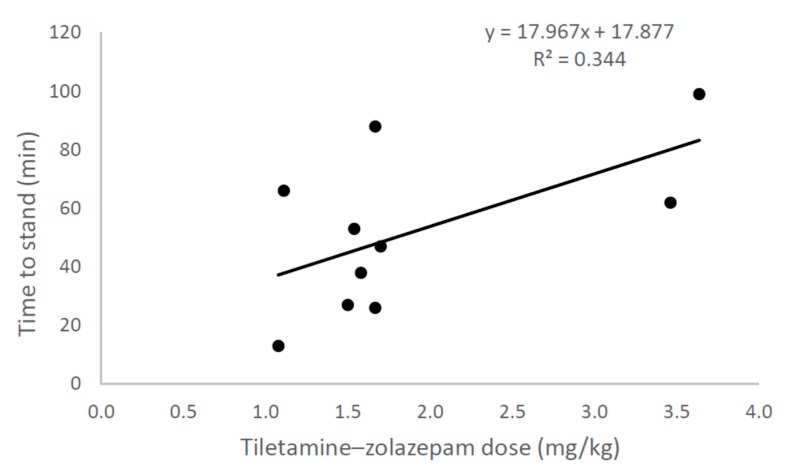
Ten ground-netted Bennett’s wallabies (*Notamacropus rufogriseus*) were lightly sedated using tiletamine–zolazepam (500 mg powder; Zoletil^®^; Virbac, NSW, Australia) using variable doses not exceeding 4 mg/kg. The results show that time to recovery (standing) were positively related to dosage, but there was large variability at lower doses, with some wallabies given low doses taking about 60–90 min to recover (the same recovery time as those wallabies that were given the highest doses).

The mean processing times for chemically immobilised and physically restrained wallabies was 14 min (range = 8–26 min) and 11 min (range = 4–20 min), respectively. The mean dose of tiletamine–zolazepam was 1.9 mg/kg (range = 1.1–3.6). Doses varied due to the initial use of a relatively high dose (c. 3.5 mg/kg), which was subsequently reduced to c. 1.5 mg/kg due to prolonged recoveries (>60 min), and due to body mass being estimated for some individuals, rather than measured, before dosing. The mean time to a sedated animal raising its head was 39 min (range = 12–85 min). The mean time to recovery (i.e., standing) was 52 min (range = 13–99 min). Time to recovery was positively related to the dose given to sedated wallabies, but there was large variability at lower doses, with some individuals given low doses taking about 60–90 min to recover (Figure 4).

In addition to wallabies, we also trapped four brushtail possums (i.e., 10% of ground-netted animals were non-target species). We euthanased captured possums following protocols approved by the Manaaki Whenua–Landcare Research (MWLR) Animal Ethics Committee (i.e., blunt trauma). In addition to welfare impacts, the capture of non-target possums also reduced the availability of nets for wallabies, and hence reduced cost-effectiveness of ground-netting.

### 3.4. Wallaby Capture Costs

Wallaby capture costs include helicopter time (including ferry time) and support personnel for net-gunning, and one ground trapper and support personnel for ground-netting. Per captured wallaby, net-gunning cost NZ$1045 (*n* = 11) and ground-based netting cost NZ$745 per captured wallaby (*n* = 36), meaning that net-gunning was 40% more expensive (Table 1). Corrected for mortalities at time of capture, net-gunning cost NZ $1149 per live wallaby (*n* = 10), whereas ground-based netting cost NZ $893 per live wallaby (*n* = 30), meaning that net-gunning was 29% more expensive. The cost per GPS-collared wallaby was NZ $1149 and NZ $1030 for net-gunning (*n* = 10) and ground-based netting (*n* = 26), respectively, meaning that net-gunning was 12% more expensive.

### 3.5. Red Deer Demography

We captured a total of 63 red deer, including 29 deer that were darted, 30 deer that were netted, and four deer that were initially darted but subsequently netted. We classified the age of captured deer as adults (>24 months), sub-adults (12–24 months), and calves (<12 months). The sex:age ratio of those captured was: 21 adult females, 19 adult males, three sub-adult females, four sub-adult males, 10 female calves, and six male calves.

In order to obtain body mass estimates for red deer at the study sites, we weighed a total of 49 dead deer. These animals were either recovered post-poisoning or were collared animals that moved significant distances from the study site and were subsequently shot and killed by the researchers. These deer were weighed using an on-board weighing system with digital readout in the helicopter (Onboard Systems, Vancouver, Washington, WA, USA; www.onboardsystems.com/). This gave us relatively coarse body mass estimates, as the scales were set to c. ± 10 kg. The mean body masses of deer at the Clarence West/Waiautoa site were 70 kg for adult females (range 50–90 kg; *n* = 11), 95 kg for adult males (range 90–100 kg; *n* = 3), 62 kg for sub-adult females and males (range 40–80 kg; *n* = 6), and 44 kg for male and female calves (range 30–60 kg; *n* = 9). The mean body masses of deer at the Upper Awatere South site were 104 kg for adult females (range 90–110 kg; *n* = 9), 148 kg for adult males (range 110–190 kg; *n* = 5), 110 kg for a sub-adult male (*n* = 1), and 66 kg for male and female calves (range 60–70 kg; *n* = 5).

### 3.6. Helicopter Net-Gunning Deer

We decided that net-gunning deer was preferable to darting in one block at Clarence West/Waiautoa because of its proximity to tall vegetation, which darted deer could potentially run into and not be found. At the remaining sites deer were netted opportunistically depending on terrain, habitat, and size of the animal.

A total of 11 adult females, three sub-adults, and 16 calves were netted (Table 1); four deer escaped from nets that had been poorly fired or had become hung up on vegetation. For 16 deer where video footage recorded chases, the mean CT was 2 min (range = 1–5 min). Of the 14 captures where the entire capture to release was recorded, the mean time spent from capture to release was 11 min (range = 6–19 min). Most of this time was spent untangling (removing) the net from around the deer: therefore, the main variation in processing time was due to how long it took to untangle a deer.

Of the 30 netted deer, three were euthanased due to capture injury resulting from falls (one broken lower jaw, one suspected broken neck, one suspected broken back). Injured deer were euthanased by either intravenous pentobarbitone administered by the veterinarian (*n* = 1) or a rifle shot to the head (*n* = 2). All three euthanased deer sustained injuries as a result of falls once netted that were out of control of the capture team. Other deer sustained bruising and knocks and were administered meloxicam for relief from inflammation and pain at a dose of 0.5 mg/kg. After c. 4 months of monitoring, there were no post-capture deaths from any deer collared and released from nets, as revealed by VHF mortality collars.

### 3.7. Helicopter Darting for Red Deer

We darted a total of 33 deer (Table 1). Of these, 29 became recumbent after a single dart, and four deer that did not become recumbent were subsequently net-gunned. As net-gunning was used as a secondary capture method in the event of a darted animal not becoming recumbent, no darted deer escaped; however, without net-gunning as a back-up, or firing a second dart, some deer may have escaped. For another five deer, chases were abandoned after several minutes before a capture attempt was made when the likelihood of a successful darting or net gun shot was considered to be low. For 24 deer where video footage recorded chases, the mean CT was 3 min (range = 1–9 min). Mean IT was 3 min (range = 2–12 min), and mean RT was 11 min (range = 5–21 min). The mean time of the full procedure was 15 min (range = 8–30 min).

Unlike net-gunning, there were no capture injuries sustained through darting; however, there was a single deer (an adult male) that was found dead with a mortality time 3.5 days post-capture (electronic recording of time-to-death). We strongly suspect this may be the result of capture myopathy [42], but because the carcass was not recovered until 3 months after death, we could not confidently determine the actual cause of death: nevertheless, we record it as a capture-related mortality here.

### 3.8. Red Deer Capture Costs

We estimated the mean capture costs for deer by tallying all staff and veterinary costs and associated helicopter time (including ferry, search and capture time) and dividing that total by the number of deer captures obtained by each capture method. Where deer were darted and subsequently netted (*n* = 4), we have allocated that cost to darting, as a veterinarian was present and the drug administered, which is the main difference in cost to physical capture alone.

The helicopter search and capture times were comparable for both methods (mean for net-gunning = 57 min, range = 20–80 min; mean for darting = 66 min, range = 20–105 min); however, there are additional costs associated with drugs and having a veterinarian present when darting. The mean cost of capturing a deer via net-gunning was NZ$2011, while the mean cost of capturing a deer via darting was NZ$2596, meaning that darting was 30% more expensive (Table 1). Corrected for mortalities within 14 days of capture, the mean cost would increase to NZ$2234 and NZ$2677, respectively, meaning that darting was 20% more expensive.

## 4. Discussion

All four capture methods assessed in our study were effective, with variable animal welfare impacts and cost-effectiveness. The least effective method was net-gunning for wallabies due to many animals (54%) escaping. The most effective method was helicopter darting for red deer, with zero escapes and a mortality rate of 3% at 14 days post-capture, but this method was the most expensive (NZ$2677 per surviving deer) of those trialled in our studies. The importance of non-target captures in our study is arguably not high, with only one capture method recording non-target impacts (ground-netting). The only non-target species captured was another invasive mammal (brushtail possum) that is commonly lethally controlled to protect conservation and production values [43,44].

### 4.1. Net-Gunning

To our knowledge this is the first study to report the use of helicopter net-gunning for any marsupial species. The technique did not appear to be ideally suited to wallabies, with about half (54%) the targeted individuals escaping the net before they could be manually restrained. This compares to similar escape rates for preliminary trials in similar-sized mammals (e.g., 55% for coyotes (*n* = 22) [29]) in the small number of studies that have reported such data. However, at the Grampians Station, a smaller percentage of netted wallabies (33%) evaded capture personnel, albeit only based on six attempts. The Grampians was the most open of the study sites and therefore provided wallabies with the least escape terrain/habitat.

The nets we used were not specifically designed for wallaby capture and wallabies often avoided becoming tangled in them. We believe that net-gunning may be an effective capture method for macropods if a net with appropriate dimensions (net and mesh size) is used in open habitats. The appropriateness of net dimensions would require field testing, but we speculate that nets with a slightly larger mesh might allow the heads and large feet of macropods to go through the holes more easily than the 4″ mesh we used. This might mean they become tangled more quickly and therefore travel a shorter distance from where they were netted.

**Figure 5:**
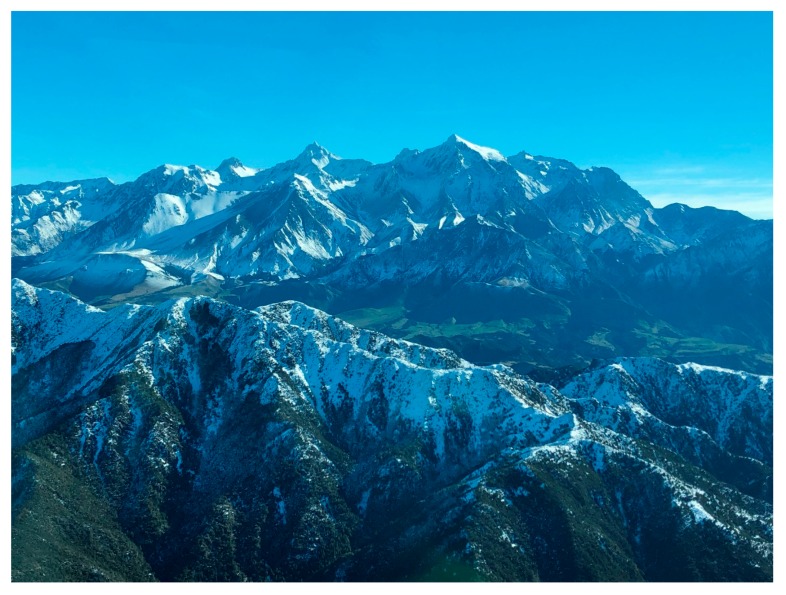
The steep, rugged terrain of Clarence Valley, south Marlborough, New Zealand, where red deer (*Cervus elaphus*) were net-gunned from a helicopter in 2019. The Waiautoa capture block, Seaward Kaikōura Range, is in the foreground, the Clarence River behind it, and the Clarence west capture block, Inland Kaikōura Range, is seen in the background. The type of terrain shown can play an important role in causing injury-related capture mortalities and the prescription of suitable capture methods.

Net-gunning was more effective for red deer, with a lower escape rate of 13%. Many studies describing net-gunning for other deer species (e.g., white-tailed deer, *Odocoileus virginianus*) do not report escape rates [35,45], making comparison difficult. The mortality rate of 10% at the time of capture was unsurprising given the steep terrain the study was undertaken in (Figure 5). All three mortalities were caused by traumatic injuries when deer fell. Similar traumatic injuries have been reported as occurring (but at lower frequencies) in long-term studies of helicopter net-gunning for North American deer. For example, 0.4% of white-tailed deer suffered broken legs in the USA [45], despite occurring in terrain less steep than in our study of red deer. We did not assess non-terminal injuries, but we suspect that these would have been higher than recorded traumatic injuries that led to euthanasia. To minimise the risk of injury, we recommend avoiding netting deer in the steepest terrain, or, if possible, using darting.

Sample sizes used in our study were smaller than those that would be required for reporting the frequency of outcomes with reasonable statistical confidence [46], but this is a necessary trade-off when performing preliminary research that may impose animal welfare impacts with ethical oversight. Studies that have reported several years of operational use of wildlife capture techniques (e.g., *n* = 3350 for net-gunning of white-tailed deer [45]) are better able to accurately quantify adverse event frequency and variables associated with their occurrence.

### 4.2. Ground-Based Netting for Wallabies

Ground netting was the most cost-effective of the four capture methods assessed, but it also produced the highest mortality rate at the time of capture (17%) and was the only capture method to have non-target impacts (brushtail possums), albeit at a low percentage (10%). Also, one of the five recorded post-capture mortalities was probably the result of capture myopathy, as GPS data showed it died shortly after capture. Macropods are known to be highly susceptible to this metabolic disease [12,13,14]; however, a maximum of three (probably two) wallabies are likely to have died from capture myopathy, with the remainder dying from some form of physical injury (strangulation or nerve damage and subsequent euthanasia).

Ground netting is most efficacious if nets are set at wallaby runs along fence lines. If fence lines occur at low densities in a study area, as is often the case on large high-country stations, there is a temptation to set nets along steep sections of fence lines in order to deploy enough nets to maximise capture opportunities. Although mortality is always a possibility with any capture method, we suspect that setting nets along steep fence lines probably contributed to at least three mortalities at the time of capture (primarily due to strangulation). We recommend, insofar as is possible, avoiding steep sections of fence lines for setting nets.

The three captures caught on camera showed that wallabies were trapped in the evening. Even with an early dawn check of nets, these three wallabies were confined for >12 h. We suspect it was not coincidental that the wallaby confined for the greatest period (>19 h) had the lowest recorded body temperature (33.6 °C), was unresponsive when found in the net, and subsequently died. Therefore, we recommend setting no more nets than can be checked, including any captured wallabies processed, by mid-morning at the latest. As a guide, we suggest 30–50 nets may be suitable for a capture crew of two to three personnel, depending on the density of wallabies in the area being trapped.

Our study is not the first to use ground-based nets with a drawstring for netting of macropods. Eastern grey kangaroos (*Macropus giganteus*) have been trapped in Australia using a similar design to that used in our study (i.e., a unidirectional net with a drawstring that is suspended from a metal frame and set along fences) [22,47]. Key differences of this design included a team of up to six people driving kangaroos towards the fence line and manually operating the drawstring if a kangaroo entered the net (as opposed to our design, which drew tight when pressure was exerted by the wallaby at the end of the net; Figure 2b). A modified design [47] captured a mean of two wallabies per morning using two to five nets in 10 trap mornings. These authors report that the trapping and handling process was so rapid that the risk of capture myopathy was minimal (no further details on animal welfare were provided). Similarly, the trapper we employed in our wallaby study had previously used ground-based nets to capture Bennett’s wallabies for international zoological gardens in the early 1990s, but no data on animal welfare were collected at this time.

### 4.3. Helicopter Darting

Helicopter darting, using thiafentanil sedation, was a highly effective capture method for red deer, with zero escapes and only one suspected mortality at the time of capture. However, the technique was not perfect, and 12% of darted deer did not become recumbent after darting and required net-gunning as a secondary capture method.

Helicopter darting has been widely used to capture other deer species, such as desert mule deer (*Odocoileus hemionus crooki*) in the USA [48], and reindeer (*Rangifer tarandus tarandus*) in Scandinavia [49]. However, helicopter darting poses some unique challenges, in that it is expensive per hour of use, can be dangerous for staff, and can impose considerable animal welfare impacts [40].

Many of these animal welfare risks can be mitigated using highly potent and fast-acting pharmacological agents. Thiafentanil (often used in combinations) has become an increasingly popular sedative for remote chemical immobilisation of wildlife [31,32,50,51,52,53]. The IT recorded with the use of thiafentanil (mean of 3 min) were short when compared to deer helicopter darting methods using non-opioid pharmacological regimens. For example, mean IT for helicopter darting of reindeer using medetomidine-ketamine [49]. Mean RT values for helicopter darting were also lower (11 min) than for many other published deer darting methods (e.g., c. 72 min for reindeer) [49]. Consequently, the duration of stress imposed by the thiafentanil darting method was considerably less than for many other helicopter darting techniques.

### 4.4. Comparing Physical and Chemical Restraint

For both Bennett’s wallabies and red deer we assessed at least one method that did not use chemical restraint (net-gunning and ground-based netting with physical restraint) and one that did (ground netting and darting). Although all methods we used had some impacts on the welfare of animals, those associated with physical restraint, especially ground-netting wallabies and net-gunning deer, were somewhat higher than using chemical restraint. For red deer, chemical immobilisation was associated with fewer traumatic injuries (bruising, rolling, flight response) and fewer mortalities. Physical restraint is usually quicker than chemical restraint, so may be better for species less inclined to capture myopathy.

However, other research has suggested that chemical immobilisation may offer an effective preventive measure for limiting myopathy-related deaths in macropods [13]. Qualitatively, lightly sedating ground-netted wallabies seemed to be helpful in reducing stress for animals and was also less stressful for the handlers. It may also have reduced or prevented the prevalence of capture myopathy, as neither of the wallabies that were inferred to have died as a result of capture myopathy were sedated. Although chemical immobilisation may be less stressful during capture and handling, non-reversible regimes such as tiletamine–zolazepam are often associated with extended RT, placing sedated animals at risk of depredation [54]. Reversible, or partially reversible regimes may reduce these risks, but their use must be practical and permissible for researchers that do not have a veterinarian as part of the capture crew [55].

Cost-effectiveness of each approach was mixed, with net-gunning (which used physical restraint) being the more expensive method for Bennett’s wallabies and the less expensive method for red deer. Eliminating the expense associated with using a helicopter, the efficacy and cost-effectiveness of physically restraining vs. lightly sedating ground-netted wallabies were comparable, with only a slight increase in cost for purchasing a small quantity of tiletamine–zolazepam. Our results do not offer an opportunity to compare stress levels between capture methods as we did not attempt to quantify levels of blood stress markers.

## 5. Conclusions

We present our case studies as examples of how to assess and refine newly developed capture methods. There is suspicion in wildlife research that many authors omit or under-report adverse events that occur during their studies to preserve the reputation or social licence of their work [56]. This trend may have led to a culture where few researchers publish methods ‘that don’t work’ and mistakes are consequently repeated [19]. Some researchers have made commendable efforts to provide full transparency on the number and cause of mortalities in their studies [35,57,58] and we have continued this approach. Other currently used capture methods that may be assessed to allow comparison with the methods we studied include ground-based darting (both species) and trapping (deer).

Our results suggest that net-gunning is relatively inefficient for wallaby capture in areas with little open habitat, but that the other three capture methods assessed were effective. Two methods, ground-netting wallabies and net-gunning deer, had comparatively higher animal welfare impacts compared with darting. Our results do not provide clear guidance for which techniques are ‘better’, which will depend on priorities regarding impacts imposed on non-captured and non-target animals, as well as cost. The transparent approach to reporting of capture outcomes presented here will facilitate the identification of ‘best practice’ techniques for capturing invasive mammals in New Zealand environments.

## Figures and Tables

**Table 1:** Number of animals captured (*n*), proportion (P) of adverse animal welfare events and costs for capture techniques applied to Bennett’s wallaby (*Notamacropus rufogriseus*) and red deer (*Cervus elaphus*) for telemetry studies in South Island, New Zealand, 2018–2019. Numbers in parentheses represent 95% confidence intervals for proportions.

Species	Technique	*n*	P (Escape)	P (Capture Mortality)	P (Non-Target Capture)	Cost Per Captured Animal	Cost Per Surviving Animal
Bennett’s wallaby	Net-gun	24	0.54	0.04	0	NZ$1045	NZ$1149
			(0.34–0.74)	(0–0.12)			
	Ground-net	36	0.03	0.17	0.10 (0.01–0.19)	NZ$745	NZ$893
			(0–0.08)	(0.04–0.29)			
Red deer	Net-gun	30	0.13	0.10	0	NZ$2011	NZ$2234
			(0.01–0.25)	(0–0.21)			
	Darting	33	0.00	0.03	0	NZ$2596	NZ$2677
			–	(0–0.09)			
